# Alleviation of salinity stress by EDTA chelated-biochar and arbuscular mycorrhizal fungi on maize via modulation of antioxidants activity and biochemical attributes

**DOI:** 10.1186/s12870-024-04753-x

**Published:** 2024-01-23

**Authors:** Ping Huang, Shoucheng Huang, Yuhan Ma, Subhan Danish, Misbah Hareem, Asad Syed, Abdallah M. Elgorban, Rajalakshmanan Eswaramoorthy, Ling Shing Wong

**Affiliations:** 1 College of Chemistry and Materials Engineering, Anhui Science and Technology University, Fengyang, Anhui 233100 China; 2 College of Life and Health Science, Anhui Science and Technology University, Fengyang, Chuzhou, Anhui 233100 China; 3 Department of Soil Science, Faculty of Agricultural Sciences and Technology, Bahauddin Zakariya University, Multan, Punjab 60000 Pakistan; 4 Department of Environmental Sciences, The Woman University Multan, Multan, Punjab 60000 Pakistan; 5 Department of Botany and Microbiology, College of Science, King Saud University, P.O. 2455, Riyadh, 11451 Saudi Arabia; 6 Center of Excellence in Biotechnology Research, King Saud University, Riyadh, Saudi Arabia; 7 Department of Biochemistry, Centre of Molecular Medicine and Diagnostics (COMMAND), Saveetha Dental College and Hospitals, Saveetha Institute of Medical and Technical Sciences (SIMATS), Chennai, 600077 India; 8 Faculty of Health and Life Sciences, INTI International University, Putra Nilai, Negeri Sembilan, Nilai, 71800 Malaysia

**Keywords:** Activated carbon, EDTA-chelated biochar, Maize, AMF, Antioxidants, Gas exchange

## Abstract

Salinity stress adversely affects agricultural productivity by disrupting water uptake, causing nutrient imbalances, and leading to ion toxicity. Excessive salts in the soil hinder crops root growth and damage cellular functions, reducing photosynthetic capacity and inducing oxidative stress. Stomatal closure further limits carbon dioxide uptake that negatively impact plant growth. To ensure sustainable agriculture in salt-affected regions, it is essential to implement strategies like using biofertilizers (e.g. arbuscular mycorrhizae fungi = AMF) and activated carbon biochar. Both amendments can potentially mitigate the salinity stress by regulating antioxidants, gas exchange attributes and chlorophyll contents. The current study aims to explore the effect of EDTA-chelated biochar (ECB) with and without AMF on maize growth under salinity stress. Five levels of ECB (0, 0.2, 0.4, 0.6 and 0.8%) were applied, with and without AMF. Results showed that 0.8ECB + AMF caused significant enhancement in shoot length (~ 22%), shoot fresh weight (~ 15%), shoot dry weight (~ 51%), root length (~ 46%), root fresh weight (~ 26%), root dry weight (~ 27%) over the control (NoAMF + 0ECB). A significant enhancement in chlorophyll a, chlorophyll b and total chlorophyll content, photosynthetic rate, transpiration rate and stomatal conductance was also observed in the condition 0.8ECB + AMF relative to control (NoAMF + 0ECB), further supporting the efficacy of such a combined treatment. Our results suggest that adding 0.8% ECB in soil with AMF inoculation on maize seeds can enhance maize production in saline soils, possibly via improvement in antioxidant activity, chlorophyll contents, gas exchange and morphological attributes.

## Introduction

Salinity stress is a significant abiotic factor that negatively impacts crop growth [1] and agriculture productivity resulting in development of food security issue [2–4]. High concentrations of salts, especially sodium chloride, in the soil or water can hinder plant development and result in various adverse consequences [5, 6]. These detrimental effects include reduced water uptake due to osmotic imbalance, ion toxicity that disrupts essential nutrient balance [4, 7], impaired nutrient uptake, stunted growth, increased oxidative stress, altered metabolism, and diminished crop yield and quality [8–11]. Excess salts hinder the absorption of water by plants, leading to dehydration and water stress [12, 13]. Furthermore, the disturbed nutrient balance and reduced availability of essential nutrients exacerbate the problem [14–17]. Inoculation of arbuscular mycorrhizal fungi (AMF) is considered an effective technology to address this issue.

Through their symbiotic associations with the roots of most crop species, AMF create mycorrhizal networks that offer multiple benefits to plants facing salinity challenges [18]. One essential advantage is the improved nutrient uptake facilitated by the extensive hyphal network, allowing plants to access vital nutrients like phosphorus despite salinity [19, 20]. Furthermore, AMF aid in maintaining osmotic balance within plant cells, preventing excessive water loss and promoting water absorption from the soil [21]. Additionally, these beneficial fungi help regulate the uptake and transport of toxic ions, such as sodium and chloride, reducing their harmful accumulation in plant tissues and mitigating ion toxicity [22]. AMF also trigger the plant’s antioxidant defense system, mitigating the harmful effects of oxidative stress caused by salinity-induced reactive oxygen species (ROS) [23].

On the other hand, biochar, a type of charcoal produced from biomass through pyrolysis, has gained attention for its potential to mitigate salinity stress in agricultural systems [24]. The addition of biochar to saline soils can enhance soil water-holding capacity and reduce water evaporation, thereby alleviating water stress for plants [25]. This is achieved through the porous structure of biochar, which can retain water and nutrients, making them more available to plants. Moreover, biochar can facilitate ion exchange and decrease soil salinity [26]. It possesses a high cation exchange capacity (CEC), enabling it to adsorb and retain excess salts like sodium, thus reducing their presence in the soil and minimizing the harmful effects on plant roots [27]. Additionally, the application of biochar can stimulate microbial activity in the soil [28]. Beneficial soil microorganisms, such as mycorrhizal fungi, thrive in the presence of biochar and can further aid in plant nutrient uptake and stress tolerance [29].

The current study aims to investigate the effects of EDTA-chelated biochar and AMF on maize (*Zea mays* L.) under salinity stress conditions. While both biochar and AMF have individually shown potential in mitigating salinity stress, their combined application and the use of EDTA-chelated biochar as a salinity alleviator in maize cultivation remain relatively unexplored. The research seeks to fill this knowledge gap and provide novel insights into the interactions between EDTA-chelated biochar and AMF, evaluating their combined impact on maize growth, nutrient uptake and physiological responses. It is hypothesized that combined application of EDTA-chelated biochar and AMF might effectively minimize salinity adverse effects on maize.

## Materials and methods

### Biochar

To produce sugar syrup waste biochar, the initial step involves collecting and thoroughly drying the sugar syrup waste to eliminate all moisture. Next, the dried waste is combined with sulfuric acid [30]. To create the biochar, the mixture is subsequently heated to approximately 400 ± 15 °C in an oxygen-free environment, like a pyrolysis reactor. The characteristics of biochar include: pH = 8.15; EC*e* (dS/m) = 5.05; Ash Content (%) = 30; Volatile Matter (%) = 20; Fixed carbon (%) = 50; Total Nitrogen (%) = 0.11; Total Phosphorus (%) = 0.49; Total Potassium (%) = 0.41; Surface area (m²/g) = 300 and CEC (meq./100 g) = 425.

### EDTA

The ethylenediaminetetraacetic acid (EDTA) utilized in the research was procured from Sigma Aldrich certified local market dealer. The obtained EDTA is classified as an ACS reagent, signifying a high purity grade, with a minimum purity of 99.4% and a maximum purity of 100.6%. It was provided in powder form and is associated with the following specific details: Product Number E9884, Batch Number BCCJ0200, Brand SIAL, and CAS Number 60-00-4.

### Chelated biochar preparation

The biochar was mixed thoroughly with EDTA dissolved in water at a concentration of 0.1 M or higher. After thorough mixing, the EDTA-chelated biochar (ECB) was left to dry completely before its application as a soil amendment.

### Arbuscular mycorrhizal fungi (AMF)

To introduce arbuscular mycorrhizal fungi (AMF) into the soil, a commercially available inoculum called Clonex® Root Maximizer was utilized. This inoculum primarily consisted of Glomus species and contained approximately 158 propagules per gram. In order to ensure optimal colonization, a quantity of 2.5 g of the inoculum was mixed with biochar (BC) according to the research treatment plan [31].

### Treatment plan

There were 5 levels of EDTA chelated biochar (ECB) i.e., control (0ECB), 0.2, 0.4, 0.6 and 0.8% ECB, applied under saline soil (5.74 dS/m)  with and without AMF. All the treatments were applied in completely randomized design following 5 replications. The pre-experimental soil and irrigation water data is provided in Table 1.

**Table 1:** Pre-experimental soil and irrigation characteristics

Soil	Values	References	Irrigation	Values	References
pH	8.22	[32]	pH	7.55	[33]
EC*e* (dS/m)	5.74	[34]	EC (µS/cm)	519	
SOM (%)	0.40	[35]	Carbonates (meq./L)	0.00	
TN (%)	0.02	[36]	Bicarbonates (meq./L)	6.09	
AP (µg/g)	3.45	[37]	Chloride (meq./L)	0.010	
EK (µg/g)	78	[38]	Ca + Mg (meq./L)	5.09	
ENa (µg/g)	516	[39]	Sodium (mg/L)	176	
Texture	Loam	[40]	TN = Total Nitrogen; AP = Available Phosphorus; EK = Extractable Potassium ENa = Extractable Sodium		

### Seeds collection and sterilization

In this study, maize (Gohar-19) was purchased from a seed dealer of Government of Punjab in Multan (30°10’26.0 N 71°28’10.7E), Punjab, Pakistan. To ensure surface sterilization, a two-step process was employed. First, the seeds were immersed in 70% ethanol for 5 min, followed by a 10-minute treatment with 5% sodium hypochlorite. Subsequently, the seeds were thoroughly rinsed with distilled water and left to soak for 24 h [41].

### Seeds sowing

Plastic pots measuring 10 inches in width and 12 inches in depth were employed to sow the maize seeds. In each pot, five seeds were initially sown. After germination, a thinning process was carried out to retain only two healthy seedlings in each pot.

### Irrigation

At the beginning of the experiment, 100mL of sterilized water was used for the initial irrigation of each pot. Subsequently, a daily water supply of 50 ml was given to each pot until the seedlings were ready for harvesting. The initial 100 ml water was added to ensure that the soil in each pot maintained a field capacity of 60%.

### Nutrients application

Hoagland solution [42] of half-strength (0.5X) was applied in soil after 5 days interval. A half-strength (0.5X) Hoagland solution was prepared by combining specific macronutrients per liter of distilled water, which included 2.5 g of calcium nitrate (Ca(NO_3_)_2_·4H_2_O), 2.5 g of potassium nitrate (KNO_3_), 0.5 g of magnesium sulfate (MgSO_4_·7H_2_O), and 0.25 g of monopotassium phosphate (KH_2_PO_4_). For the provision of micronutrients, 20 milligrams of iron (Fe) chelate (e.g., Fe-EDTA), 2 milligrams of boric acid (H_3_BO_3_), 2 milligrams of manganese sulfate (MnSO_4_·H_2_O), 2 milligrams of zinc sulfate (ZnSO_4_·7H_2_O), 0.5 milligrams of copper sulfate (CuSO_4_·5H_2_O), and 0.05 milligrams of ammonium molybdate ((NH_4_)_6_Mo_7_O_24_·4H_2_O) were included. The pH of the solution was adjusted to approximately 6.0 using either hydrochloric acid (HCl) or sodium hydroxide (NaOH) as necessary. During each application, 50 ml of Hoagland solution was administered to each treatment to prevent any nutrient stress.

### Harvesting, samples and data collection

After 35 days from the sowing date, the seedlings were harvested. Various morphological attributes, including shoot and root length, fresh and dry weights of shoot, leaves and root, were measured immediately after harvesting using a standard measuring scale and an analytical grade digital balance. Additionally, fresh leaf samples were collected and stored in liquid nitrogen to preserve them for further biochemical analysis.

### Chlorophyll determination

0.1 g of fresh leaf tissue was carefully collected from each plant and placed in individual 15 mL Falcon tubes to extract pigments. Subsequently, 10 mL of 80% acetone was added to each tube, and the samples were vigorously vortexed at maximum speed for 30 s to facilitate pigment extraction. The tubes were kept in a dark environment at room temperature for 24 h to ensure complete extraction. After the extraction period, the samples were centrifuged for 10 min at 3000 revolutions per minute to remove any remaining tissue residues. The supernatant, containing the extracted pigments, was then carefully transferred to fresh 15 mL Falcon tubes. Finally, the absorbance of the samples was measured at 663 nm and 645 nm using a UV-Vis spectrophotometer [43].

$$\text{C}\text{h}\text{l}\text{o}\text{r}\text{o}\text{p}\text{h}\text{y}\text{l}\text{l} \text{ a} \left(\frac{\text{m}\text{g}}{\text{g}}\right)=\frac{\left(12.7 \times \text{A}663\right) - \left(2.69 \times \text{A}645\right)\times \text{V}}{1000 \times \text{W}}$$

$$\text{C}\text{h}\text{l}\text{o}\text{r}\text{o}\text{p}\text{h}\text{y}\text{l}\text{l} \text{ b} \left(\frac{\text{m}\text{g}}{\text{g}}\right)=\frac{\left(22.9 \times \text{A}645\right) - \left(4.68 \times \text{A}663\right)\times \text{V}}{1000 \times \text{W}}$$

$$\text{T}\text{o}\text{t}\text{a}\text{l} \text{ C}\text{h}\text{l}\text{o}\text{r}\text{o}\text{p}\text{h}\text{y}\text{l}\text{l} \left(\frac{\text{m}\text{g}}{\text{g}}\right)= 20.2\left(\text{O}\text{D} 645\right)+8.02\left(\text{O}\text{D} 663\right)\times \text{V}/1000 \left(\text{W}\right)$$

### Electrolyte leakage

The leaves are then washed with deionized water and dried using a paper towel. The dried leaves are weighed to obtain their dry weight and then placed in separate 50 mL centrifuge tubes containing 10 mL of deionized water. These tubes are incubated in a shaking incubator at a temperature of 25 °C for approximately two hours, allowing the solution to reach equilibrium. Following the incubation period, the solution’s initial electrical conductivity (C1) is measured using a conductivity meter. Next, the samples are autoclaved for 20 min at 121 °C to effectively kill the cells and release the electrolytes present in the leaves. Once the samples have cooled down to room temperature, the final electrical conductivity (C2) is measured using a conductivity meter.

$$\text{E}\text{l}\text{e}\text{c}\text{t}\text{r}\text{o}\text{l}\text{y}\text{t}\text{e}\, \text{L}\text{e}\text{a}\text{k}\text{a}\text{g}\text{e} \left(\%\right)= (\text{C}2 - \text{C}1)/(\text{C}1)\times 100$$

### Gas exchange attributes

Leaf gas exchange attributes i.e., photosynthetic rate, net transpiration rate, and stomatal conductance, were assessed using an Infra-Red Gas Analyzer (CI-340 Photosynthesis system, CID, Inc. USA). Four wheat leaves were combined for analysis. The measurements were conducted on a sunny day, specifically between 9:36 AM and 10:45 AM [44].

### Superoxide dismutase (SOD)

The specific activity of superoxide dismutase (SOD) was determined by measuring the amount of enzyme required to reduce the rate of NBT reduction by 50%. The specific activity is expressed as EU (Enzyme Unit) per milligram of protein. A spectrophotometer was used to measure the absorbance at 560 nm [45, 46].

### Catalase (CAT) activity

The enzyme extract was combined with a reaction mixture containing 50 mM phosphate buffer at pH 7.0 and 10 mM H_2_O_2_. The absorbance of the reaction was then measured at 240 nm using a spectrophotometer. The catalase (CAT) activity was determined by utilizing the extinction coefficient of H_2_O_2_, which is 0.0394 mM^− 1^ cm^− 1^. By applying this coefficient and analyzing the change in absorbance over time, the CAT activity was calculated and expressed as EU per milligram of protein [47].

### Ascorbate peroxidase (APX) activity

A reaction mixture was prepared to evaluate the activity of ascorbate peroxidase (APX), consisting of 0.1 mM EDTA, 0.1 mM ascorbate, 0.5 mM H_2_O_2_, and the enzyme extract. The reaction was initiated by adding the enzyme extract to the mixture, and the absorbance at 290 nm was measured every 30 s for a total duration of three minutes. The enzyme activity of APX was quantified using the extinction coefficient of 2.8 mM^− 1^ cm^− 1^, which allows for the calculation of the reaction rate based on the change in absorbance at 290 nm over time [48].

### Malondialdehyde (MDA) concentration

In the experiment, 0.5 g of fresh wheat leaves was ground in 5 cc of 0.1% trichloroacetic acid. The resulting mixture was then subjected to centrifugation at 12,000 rpm for 15 min. After centrifugation, the supernatant (2 mL) was collected and mixed with 2 ml of 0.5% thiobarbituric acid. This mixture was then boiled for 30 min, followed by cooling and another round of centrifugation at 4,000 rpm for 10 min. The malondialdehyde (MDA) concentration was estimated using an extinction coefficient of 155 mM^− 1^ cm^− 1^. The MDA concentration was expressed as micromoles (µM) of MDA per gram of the sample’s fresh weight (fr wt). To determine the MDA concentration, the absorbance of the supernatant was measured at 532  nm [49].

### Ascorbic acid (AsA) concentration

To determine the ascorbic acid (AsA) content in plant tissues were ground using a pre-chilled mortar and pestle with 6% (w/v) trichloroacetic acid (TCA) on an ice bath. After homogenization, the supernatant was obtained by centrifuging the sample at 12,000 rpm for 10 min at 4 °C. Next, the DNPH reagent was prepared by dissolving 0.5% (w/v) 2,4-dinitrophenylhydrazine (DNPH) in 70% (v/v) TCA. An equal volume of DNPH reagent was added to the supernatant, and the mixture was gently mixed and incubated in the dark at room temperature for 1 h. An equal volume of 6% (w/v) thiourea in 90% (v/v) ethanol was added to the reaction mixture to reduce any interfering compounds. This extraction step was followed by incubating the mixture in the dark at room temperature for 30 min. The absorbance of the standards and solutions was measured at 520 nm using a spectrophotometer.

### Glutathione reductase (GR) activity

The reaction mixture for the glutathione reductase (GR) assay was prepared by combining 50 mM potassium phosphate buffer (pH 7.8), 0.2 mM EDTA, 0.1 mM NADPH (Nicotinamide adenine dinucleotide phosphate), and 0.5 mM oxidized glutathione (GSSG). The enzyme extract was then added to the reaction mixture and gently mixed contents. The reduction of GSSG to GSH was monitored by measuring the decrease in absorbance at 340 nm using a spectrophotometer. To determine the GR activity, EU per milligram of protein, the change in absorbance was calculated using the extinction coefficient for NADPH (6.22 mM^− 1^ cm^− 1^).

### Glutathione (GSH) activity

In the glutathione (GSH) assay, the reaction mixture was prepared by combining 0.1 M phosphate buffer at pH 7.0 with the 5,5’-dithiobis (2-nitrobenzoic acid) (DTNB) reagent. Following the reaction, the absorbance of the reaction mixture was measured at 412 nm using a spectrophotometer.

### Statistical analysis

The data were analyzed using standard statistical procedures [50]. Two-way ANOVA were performed with OriginPro 2021 software [51], followed by pairwise comparison of means using Tukey’s test at a significance level of 5%. Principal component analysis (PCA) was also carried out using OriginPro 2021.

## Results

### Shoot length, shoot fresh weight, and shoot dry weight

**Fig. 1:**
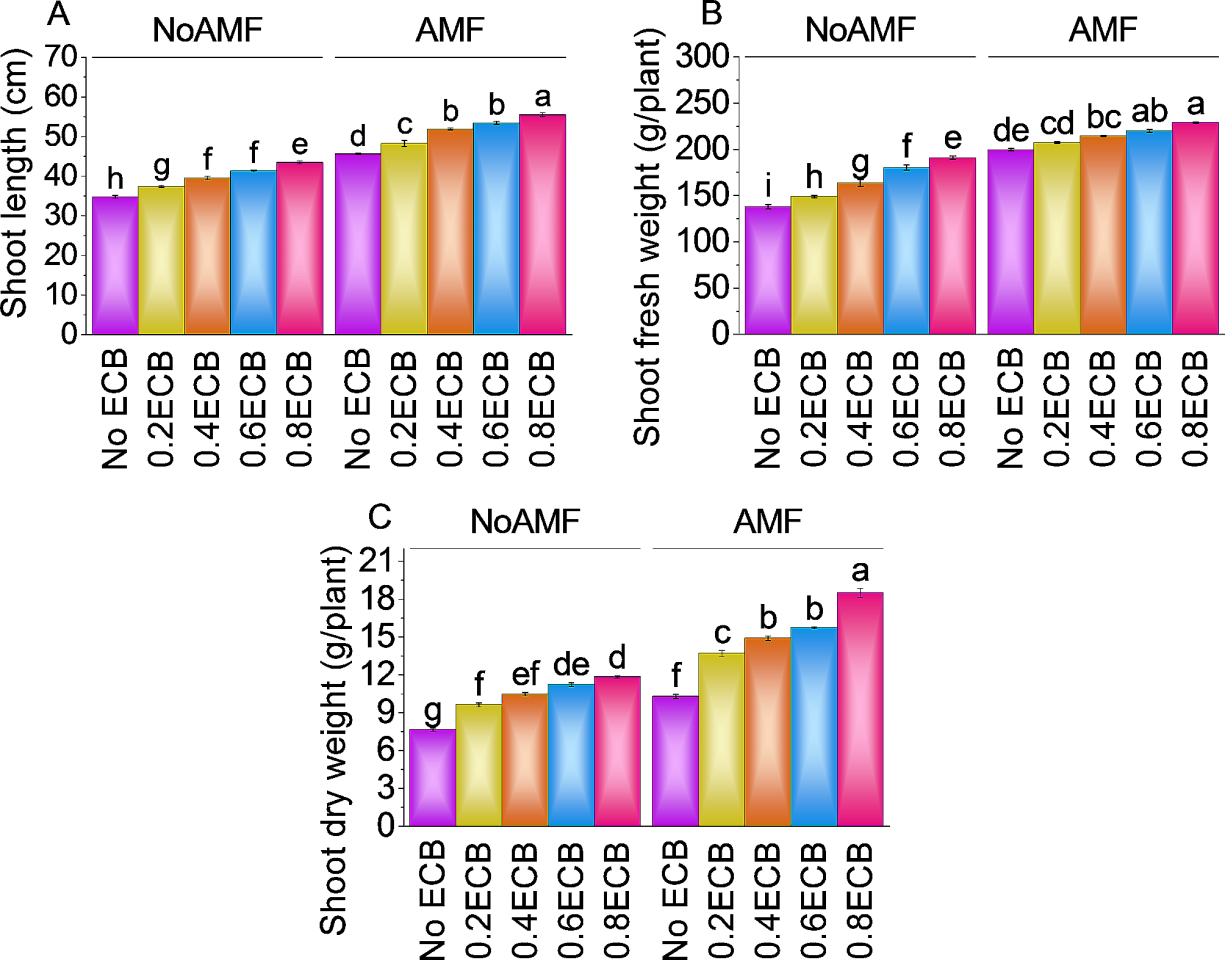
Effect of treatments on shoot length (**A**), shoot fresh weight (**B**), and shoot dry weight (**C**) of maize cultivated under NoAMF + 0ECB and AMF + 0ECB. Bars are means of 5 replicates ± SE. Difference letters on bars showed significant changes at *p* ≤ 0.05; Tukey test

The average shoot length without AMF or ECB was ~ 35 cm. Using 0.2ECB without AMF, the plants had a ~ 8% increase in shoot length over the control (NoAMF + 0ECB), averaging ~ 37 cm. Treatment 0.4ECB enhanced shoot length by ~ 14%, compared to the control with no AMF and 0ECB. Plants at 0.6 ECB had an average shoot length of ~ 41 cm, ~ 19% higher than NoAMF + 0ECB. At the highest ECB level, 0.8ECB, shoot length averaged ~ 44 cm, up ~ 25% from no AMF + 0ECB. With AMF but 0ECB, the average shoot length was 45.63 cm. After adding 0.2ECB to AMF, shoot length averaged ~ 48 cm, ~ 6% longer than the control. Adding 0.4 ECB + AMF increased shoot length by ~ 14% to ~ 52 cm. ECB levels of 0.6ECB resulted in an average shoot length of ~ 53 cm, ~ 17% longer than AMF + 0ECB. AMF + 0.8ECB increased shoot length by ~ 22%, compared to the control treatment (Fig. 1A).

When 0.2ECB was added without AMF, shoot fresh weight increased by 8.03%, compared to the control treatment. Adding 0.4ECB enhanced shoot fresh weight by 18.58%, in comparison with the control group (NoAMF + 0ECB). After raising ECB level to 0.6ECB, the plants had a mean shoot fresh weight of ~ 180 g/pot, a ~ 31% increase over the control treatment (NoAMF + 0ECB). The highest ECB dose, 0.8ECB, increased shoot fresh weight by ~ 39% in comparison to the control treatment (NoAMF + 0ECB). For AMF with 0ECB, the mean shoot fresh weight was ~ 200 g/pot. Adding 0.2ECB to the AMF-only treatment improved shoot fresh weight by ~ 4%, averaging ~ 207 g/pot. In addition, adding 0.4ECB to AMF improved shoot fresh weight by ~ 8% to ~ 214 g/pot. A mean shoot fresh weight of ~ 220 g/pot was obtained with 0.6ECB, a ~ 10% improvement over AMF + 0ECB. Finally, 0.8ECB coupled with AMF increased shoot fresh weight by ~ 15%, mean ~ 229 g/pot, over to the AMF treatment (Fig. 1B).

Without AMF and ECB, shoot dry weight averaged ~ 9 g/pot. When just 0.2ECB was given without AMF, shoot dry weight increased by ~ 11%, with a mean of ~ 10 g/pot in the control treatment. After adding 0.4ECB, the shoot dry weight improved ~ 21% to ~ 11 g/pot compared to the control group (NoAMF + 0ECB). Treatment 0.6ECB the plants had a mean shoot dry weight of ~ 11 g/pot, a ~ 30% increase over the control treatment (NoAMF + 0ECB). In the case of 0.8ECB, shoot dry weight was increased by ~ 37%. The mean shoot dry weight was ~ 12 g/pot with AMF but 0ECB. Shoot dry weight increased ~ 11% with 0.2ECB and AMF, averaging ~ 14 g/pot over AMF + 0ECB. In addition, adding 0.4ECB to AMF increased shoot dry weight by ~ 21% to ~ 15 g/pot. Increasing ECB to 0.6ECB with AMF increased mean shoot dry weight by ~ 28% to ~ 16 g/pot. Finally, AMF + 0.8ECB increased shoot dry weight by ~ 51% to ~ 19 g/pot, compared to AMF + 0ECB (Fig. 1C).

### Root length, root fresh weight, and root dry weight

**Fig. 2:**
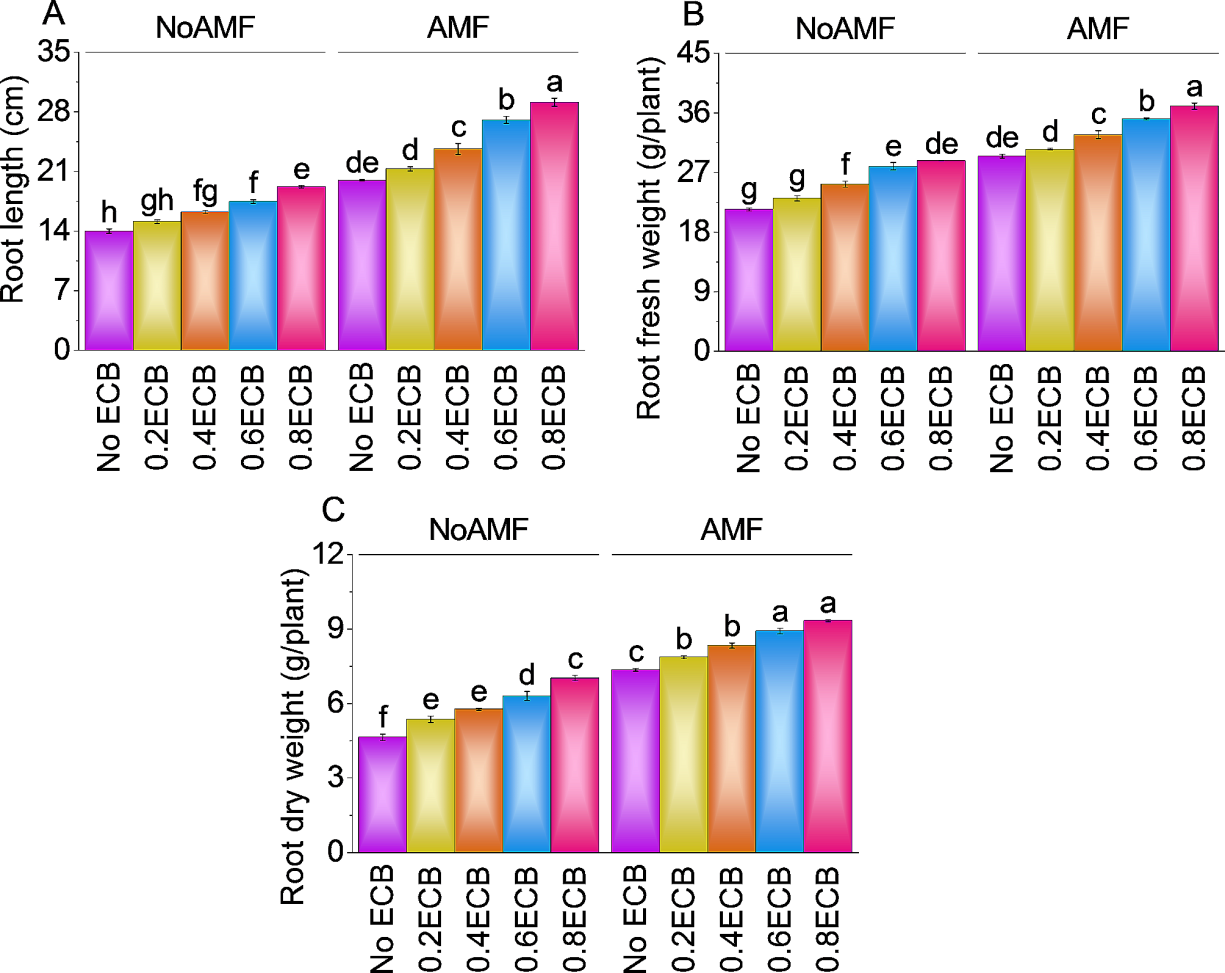
Effect of treatments on root length (**A**), root fresh weight (**B**), root dry weight (**C**), and of maize cultivated under NoAMF + 0ECB and AMF + 0ECB. Bars are means of 5 replicates ± SE. Difference letters on bars showed significant changes at *p* ≤ 0.05; Tukey test

The plants root length increased ~ 8% above the control (NoAMF + 0ECB) with a mean of ~ 15 cm when 0.2ECB was added without AMF. Additionally, adding 0.4ECB increased root length by ~ 16%, with a mean of ~ 16 cm. After adding 0.6ECB, mean root length increased by ~ 25% from (NoAMF + 0ECB). In particular, the highest examined ECB level of 0.8ECB improved root length by ~ 37%, averaging ~ 19 cm. With AMF but without ECB, root length averaged ~ 20 cm. Adding 0.2ECB to AMF increased root length by ~ 7%, averaging ~ 21 cm from the control. In comparison to AMF + 0ECB, 0.4ECB, and AMF increased root length by ~ 18% to ~ 24 cm. ECB increased to 0.6ECB leading to a mean root length of ~ 27 cm, a ~ 36% improvement over AMF + 0ECB. Finally, 0.8ECB mixed with AMF increased root length by ~ 46%, reaching ~ 29 cm (Fig. 2A).

Applying 0.2ECB treatment without AMF resulted in a significant ~ 8% increase in root fresh weight from control treatment (NoAMF + 0ECB). At 0.4ECB further enhanced the root fresh weight i.e., ~ 18% over control (NoAMF + 0ECB). The application of 0.6ECB led to a mean root fresh weight of ~ 28 g per plant, representing a notable ~ 30% increase compared to the control treatment (NoAMF + 0ECB). Notably, the highest ECB level of 0.8ECB resulted in a significant increase in root fresh weight, with a mean of ~ 29 g, corresponding to a remarkable ~ 34% increase over NoAMF + 0ECB. Combining 0.2ECB with AMF resulted in a ~ 4% increase in root fresh weight, with a mean of ~ 31 g over AMF + 0ECB. Further addition of 0.4ECB + AMF led to an 11.05% increase in root fresh weight from AMF + 0ECB. Additionally, 0.6ECB with AMF displayed a significant ~ 19% increase in root fresh weight than AMF + 0ECB. Finally, 0.8ECB combined with AMF led to a substantial increase in root fresh weight, i.e., ~ 26% compared to AMF + 0ECB (Fig. 2B).

When 0.2ECB treatment was applied without AMF, the plants exhibited a ~ 16% increase in root dry weight, compared to the no AMF + 0ECB treatment. Subsequently, adding 0.4ECB further enhanced the root dry weight to a mean of ~ 6 g, indicating a ~ 24% increase over NoAMF + 0ECB. Compared to no AMF + 0ECB, 0.6ECB improved mean root dry weight by ~ 36% to ~ 6 g. Compared to the NoAMF + 0ECB treatment, the highest ECB level, 0.8ECB, increased root dry weight by ~ 51% to ~ 7 g. With AMF but 0ECB, the mean root dry weight was ~ 7 g. Root dry weight increased ~ 7% with 0.2ECB + AMF, averaging ~ 8 g over AMF + 0ECB. Adding 0.4ECB and AMF increased root dry weight by ~ 13%. At 0.6ECB, the average root dry weight was ~ 9 g, a ~ 21% higher from AMF + 0ECB. Finally, AMF + 0.8ECB improved root dry weight by ~ 27% (Fig. 2C).

### Number of leaves, leaves fresh weight, and leaves dry weight

**Fig. 3:**
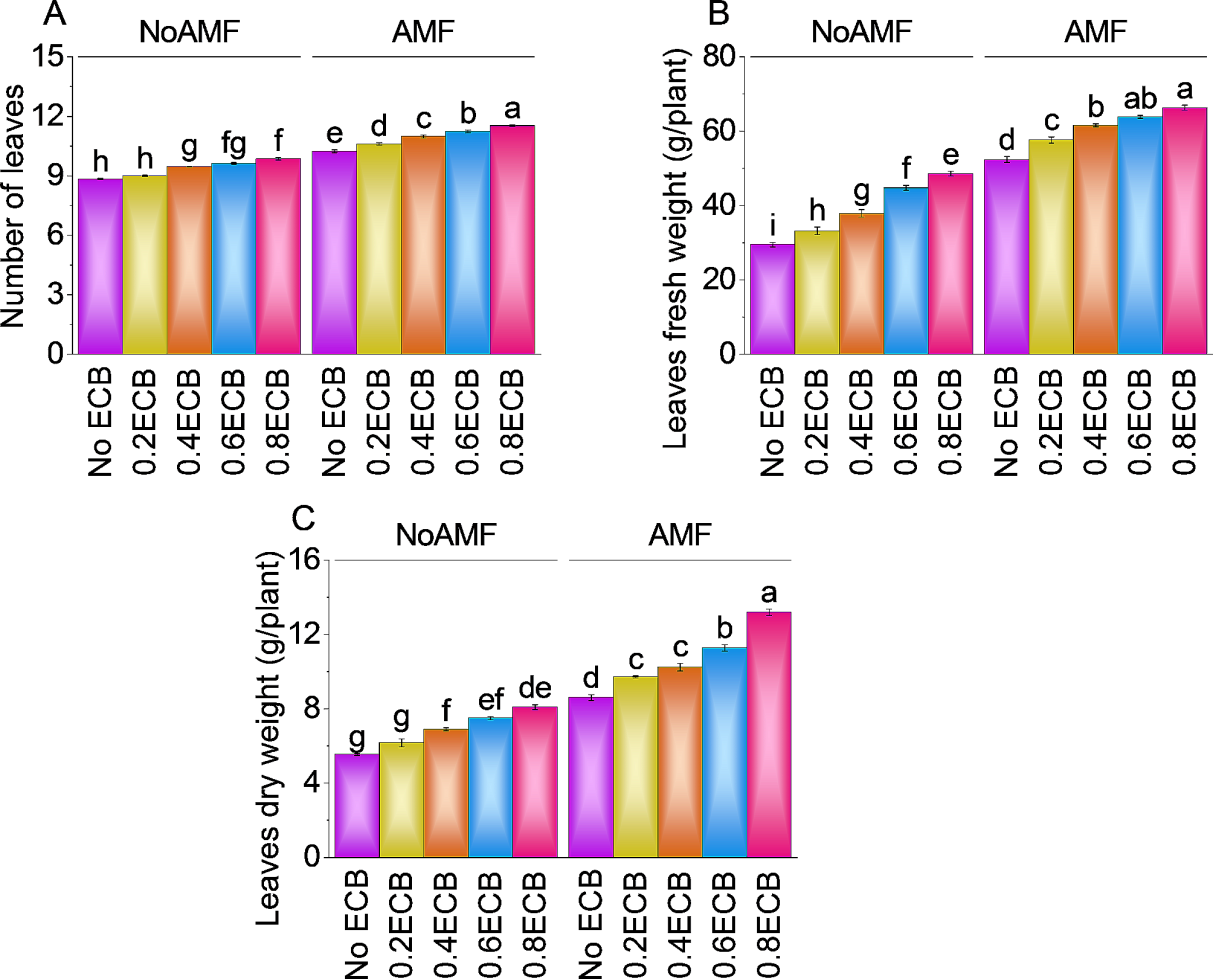
Effect of treatments on root number of leaves (**A**), leaves fresh weight (**B**) and leaves dry weight (**C**) of maize cultivated under NoAMF + 0ECB and AMF + 0ECB. Bars are means of 5 replicates ± SE. Difference letters on bars showed significant changes at *p* ≤ 0.05: Tukey test

The mean number of leaves was ~ 9 without AMF and ECB. Compared to NoAMF + 0ECB, 0.2ECB without AMF increased leaf count by ~ 2%. The addition of 0.4ECB increased leaf count by ~ 7% over NoAMF + 0ECB, with a mean of ~ 10. Compared to the NoAMF + 0ECB, 0.6ECB increased leaf number by ~ 9% to ~ 10. Notably, 0.8ECB, increased leaf number by ~ 12% from NoAMF + 0ECB, with a mean of ~ 10. AMF + ECB had a mean of ~ 10 leaves. Over the AMF + 0ECB, adding 0.2ECB to AMF increased leaf number by ~ 4%. In addition, adding 0.4ECB to AMF raised leaf number by ~ 7% to ~ 11. In comparison to NoAMF + 0ECB, increasing the ECB to 0.6ECB produced 11.24 leaves, a ~ 10% increase. Furthermore, 0.8ECB + AMF, increased leaf number by ~ 13% than NoAMF + 0ECB, with a mean of ~ 12 (Fig. 3A).

The average fresh leaf weight without AMF and ECB was ~ 30 g. However, a 0.2ECB treatment without AMF increased the leaves fresh weight by ~ 13%, comparable to the no AMF + 0ECB treatment. Addition of 0.4ECB increased leaf fresh weight by ~ 28% to ~ 38 g than NoAMF + 0ECB. After applying 0.6ECB, the mean leaves fresh weight was ~ 45 g, a ~ 52% increase above no AMF + 0ECB. At 0.8ECB, the leaf fresh weight was increased by ~ 65% from no AMF + 0ECB. With AMF + 0ECB, the mean leaf fresh weight was ~ 52 g. The leaf fresh weight increased ~ 10% with 0.2ECB + AMF treatment, averaging ~ 58 g. Adding 0.4ECB + AMF increased fresh leaf weight by ~ 18%. A mean leaves fresh weight of ~ 64 g was achieved with 0.6ECB, a ~ 22% increase from AMF + 0ECB. Finally, 0.8ECB with AMF, increased the leaf fresh weight by ~ 27%, averaging ~ 66 g (Fig. 3B).

The average dry leaf weight without AMF and ECB was ~ 6 g. A 0.2ECB treatment increased leaf dry weight by ~ 11%, with a mean of ~ 6 g, compared to no AMF + 0ECB. The addition of 0.4ECB raised the leaf dry weight by ~ 25% to ~ 7 g, compared to the NoAMF + 0ECB. AMF with 0.6ECB increased the mean leaf dry weight by ~ 35% to ~ 8 g. The 0.8ECB treatment, increased leaf dry weight by ~ 46% to ~ 8 g. Leaf dry weight averaged ~ 9 g with AMF + 0ECB. Before adding 0.2ECB to AMF, the leaves dry weight was ~ 10 g, but after adding 0.2ECB, it was ~ 13% higher. Adding 0.4ECB to AMF increased the leaves dry weight to ~ 10 g, a ~ 19% improvement over AMF + 0ECB. After increasing the ECB level i.e., 0.6 ECB, the average leaves dry weight changed significantly (~ 31%) compared to AMF + 0ECB. The highest ECB level (0.8ECB) with AMF, caused a large increase in leaves dry weight, with a ~ 53% increase over the control (Fig. 3C).

### Chlorophyll a, chlorophyll b, and total chlorophyll

**Fig. 4:**
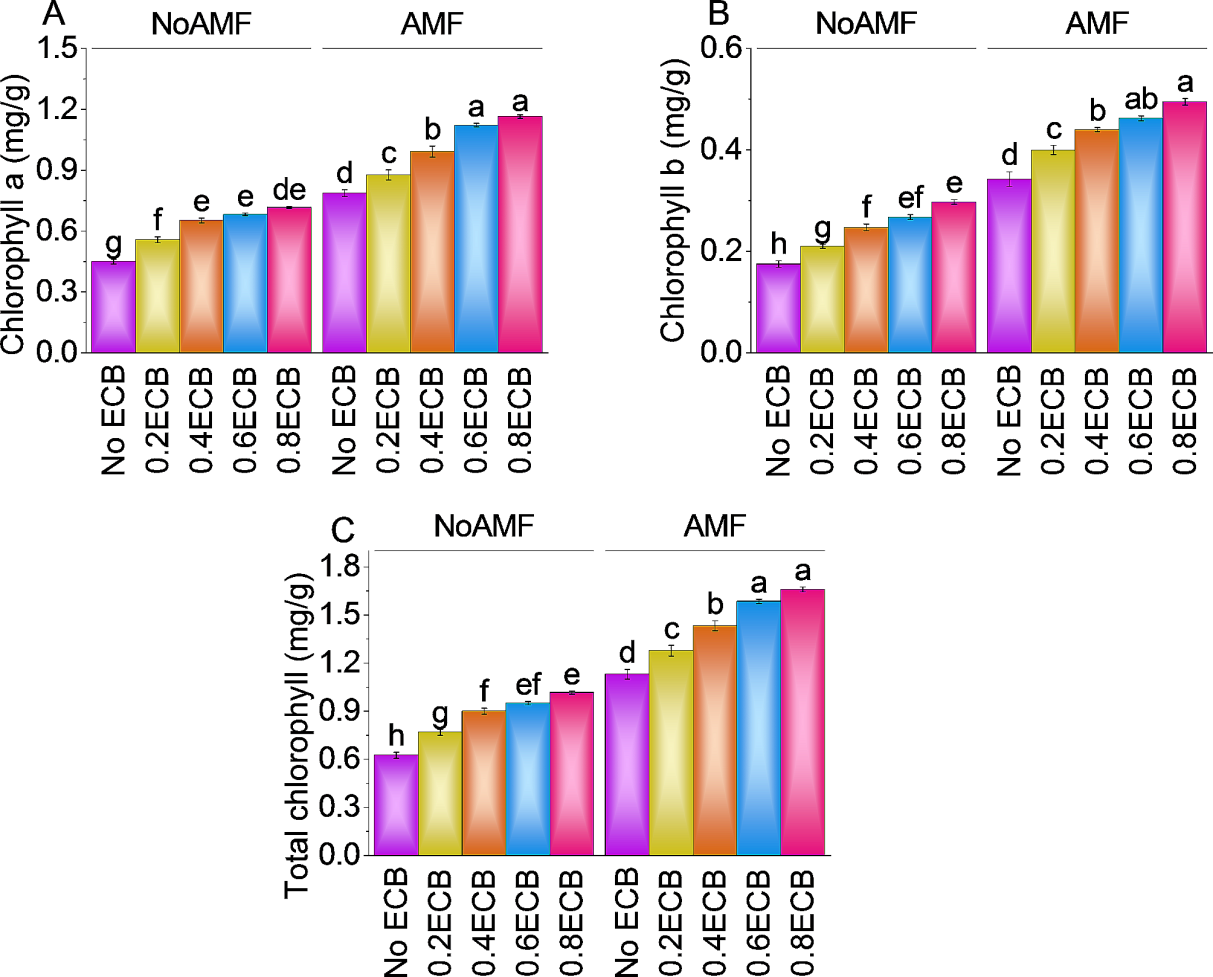
Effect of treatments on chlorophyll a (**A**), chlorophyll b (**B**) and total chlorophyll (**C**) of maize cultivated under NoAMF + 0ECB and AMF + 0ECB. Bars are means of 5 replicates ± SE. Difference letters on bars showed significant changes at *p* ≤ 0.05: Tukey test

The mean chlorophyll a content without AMF and 0ECB was ~ 0.5 mg/g. However, adding 0.2ECB without AMF increased chlorophyll a concentration by ~ 24%, with a mean of ~ 0.6 mg/g compared to the control (NoAMF + 0ECB). Adding 0.4ECB increased chlorophyll a concentration by ~ 45% compared to NoAMF + 0ECB, averaging ~ 0.7 mg/g. Adding 0.6ECB increased mean chlorophyll a concentration by ~ 52% than no AMF + 0ECB. For 0.8ECB, increased in chlorophyll a content was ~ 59% than control. AMF without ECB had a mean chlorophyll a content of ~ 0.8 mg/g. AMF + 0.2ECB increased chlorophyll a concentration by ~ 11%, with a mean of ~ 0.9 mg/g. Adding 0.4ECB to AMF increased chlorophyll a concentration by ~ 26% to ~ 1 mg/g. A mean chlorophyll a concentration of ~ 1 mg/g was noted in 0.6ECB, which was a ~ 43% increase from AMF + 0ECB. Finally, 0.8ECB, coupled with AMF, increased chlorophyll a content by ~ 48% to ~ 1 mg/g (Fig. 4A).

The mean chlorophyll b content increased ~ 20% after 0.2ECB treatment without AMF addition. Applying 0.4ECB increased chlorophyll b concentration by ~ 41% compared to NoAMF + 0ECB. It was noted that in 0.6ECB, mean chlorophyll b concentration was ~ 0.3 mg/g, which was a ~ 53% increase over no AMF + 0ECB. Notably, 0.8ECB, led to a significant increase in chlorophyll b concentration, with a mean of ~ 0.3 mg/g, corresponding to a substantial ~ 70% improvement related to the no AMF + 0ECB treatment. On the other hand, when AMF was added without ECB, the mean chlorophyll b concentration was recorded as ~ 0.3 mg/g. Adding a 0.2ECB with AMF resulted in a ~ 17% increase in chlorophyll b concentration over the control (AMF + 0ECB). Furthermore, adding 0.4ECB along with AMF enhanced the chlorophyll b concentration up to ~ 29% compared to the AMF + 0ECB. Increasing the ECB level to 0.6ECB resulted in a mean chlorophyll b concentration of ~ 0.5 mg/g, reflecting a significant ~ 35% change compared to the AMF + 0ECB. At 0.8ECB + AMF, a significant increase in chlorophyll b concentration, with a mean of ~ 0.5 mg/g, corresponding to a ~ 45% increase was noted compared to the AMF + 0ECB (Fig. 4B).

The mean total chlorophyll content without AMF or ECB was ~ 0.6 mg/g. Adding 0.2ECB without AMF improved total chlorophyll content by ~ 23%, with a mean of ~ 0.8 mg/g over the control treatment. Application of 0.4ECB increased total chlorophyll content by ~ 44% to ~ 0.9 mg/g, when compared to NoAMF + 0ECB). Comparing to the no AMF + 0ECB treatment, 0.6ECB enhanced mean total chlorophyll content by ~ 52%. The highest ECB level tested, 0.8ECB, increased total chlorophyll content by ~ 62%, in comparison with the NoAMF + 0ECB. AMF without ECB had a mean total chlorophyll content of ~ 1 mg/g. Adding 0.2ECB with AMF increased total chlorophyll content by ~ 13%. The combination of 0.4ECB and AMF improved total chlorophyll content by ~ 27%. At 0.6ECB, the mean total chlorophyll content was 1.59 mg/g, an increase of ~ 40% from AMF + 0ECB. Lastly 0.8ECB combined with AMF, enhanced total chlorophyll content by ~ 47% (Fig. 4).

### Photosynthetic rate, transpiration rate, and stomatal conductance

**Fig. 5:**
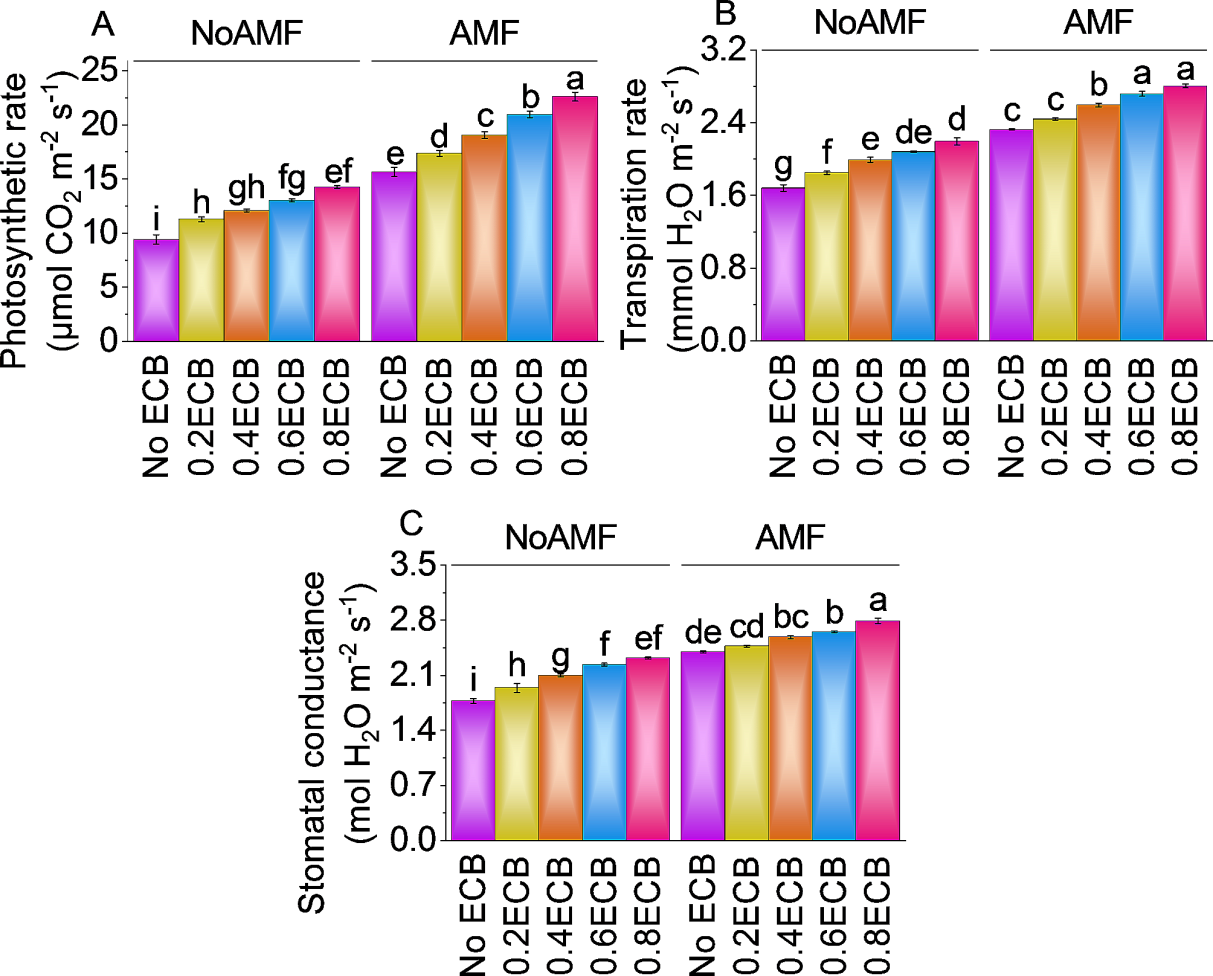
Effect of treatments on photosynthetic rate (**A**), transpiration rate (**B**) and stomatal conductance (**C**) of maize cultivated under NoAMF + 0ECB and AMF + 0ECB. Bars are means of 5 replicates ± SE. Difference letters on bars showed significant changes at *p* ≤ 0.05: Tukey test

Conversely, when AMF was present without ECB, the mean photosynthetic rate was recorded as ~ 16 µmol CO_2_ m^− 2^ s^− 1^. Adding a 0.2ECB treatment with AMF resulted in an ~ 11% increase in the photosynthetic rate than the control treatment (AMF + 0ECB), with a mean of ~ 17 µmol CO_2_ m^− 2^ s^− 1^. Furthermore, including 0.4ECB along with AMF led to a photosynthetic rate of ~ 19 µmol CO_2_ m^− 2^ s^− 1^, indicating a substantial ~ 22% increase compared to the (AMF + 0ECB) treatment. Increasing the ECB level to 0.6ECB resulted in a mean photosynthetic rate of ~ 21 µmol CO_2_ m^− 2^ s^− 1^, reflecting a significant ~ 34% improvement parallel to the (AMF + 0ECB) treatment. Finally, the highest ECB level of 0.8ECB, combined with AMF, yielded a mean photosynthetic rate of ~ 23 µmol CO_2_ m^− 2^ s^− 1^, corresponding to a remarkable ~ 45% rise above the (AMF + 0ECB) treatment (Fig. 5A).

In the absence of AMF and ECB, transpiration averaged ~ 2 mmol H_2_O m^− 2^ s^− 1^. However, adding 0.2ECB without AMF increased transpiration by ~ 10%. In addition, adding 0.4ECB increased transpiration by ~ 18%. After applying 0.6ECB, mean transpiration rate increased by ~ 24%. The highest ECB amount tested, 0.8ECB, increased transpiration by ~ 31% over no AMF + 0ECB. AMF without ECB. Adding 0.2ECB to AMF increased transpiration by ~ 5%. Compared to AMF + 0ECB, 0.4ECB + AMF increased transpiration by ~ 12%. With 0.6ECB, a ~ 17% increase in transpiration rate was noted from AMF + 0ECB. Finally, 0.8ECB + AMF, increased transpiration by ~ 21% to ~ 3 mmol H_2_O m^− 2^ s^− 1^ (Fig. 5B).

Compared to the control treatment (NoAMF + 0ECB), adding 0.2ECB without AMF increased stomatal conductance by ~ 9% to ~ 2 mol H_2_O m^− 2^ s^− 1^. It was noted that 0.4ECB increased stomatal conductance by v18% over NoAMF + 0ECB. The stomatal conductance after 0.6ECB application showed a 25.92% improvement over no AMF + 0ECB. Stomatal conductance was increased ~ 31% at 0.8ECB from no AMF + 0ECB. Adding 0.2ECB to the AMF-only treatment increased stomatal conductance by ~ 3%. Adding 0.4ECB with AMF increased stomatal conductance by ~ 8%. Under 0.8ECB + AMF stomatal conductance was enhanced by ~ 16% from AMF + 0ECB (Fig. 5C).

### Hydrogen peroxide and electrolyte leakage

**Fig. 6:**
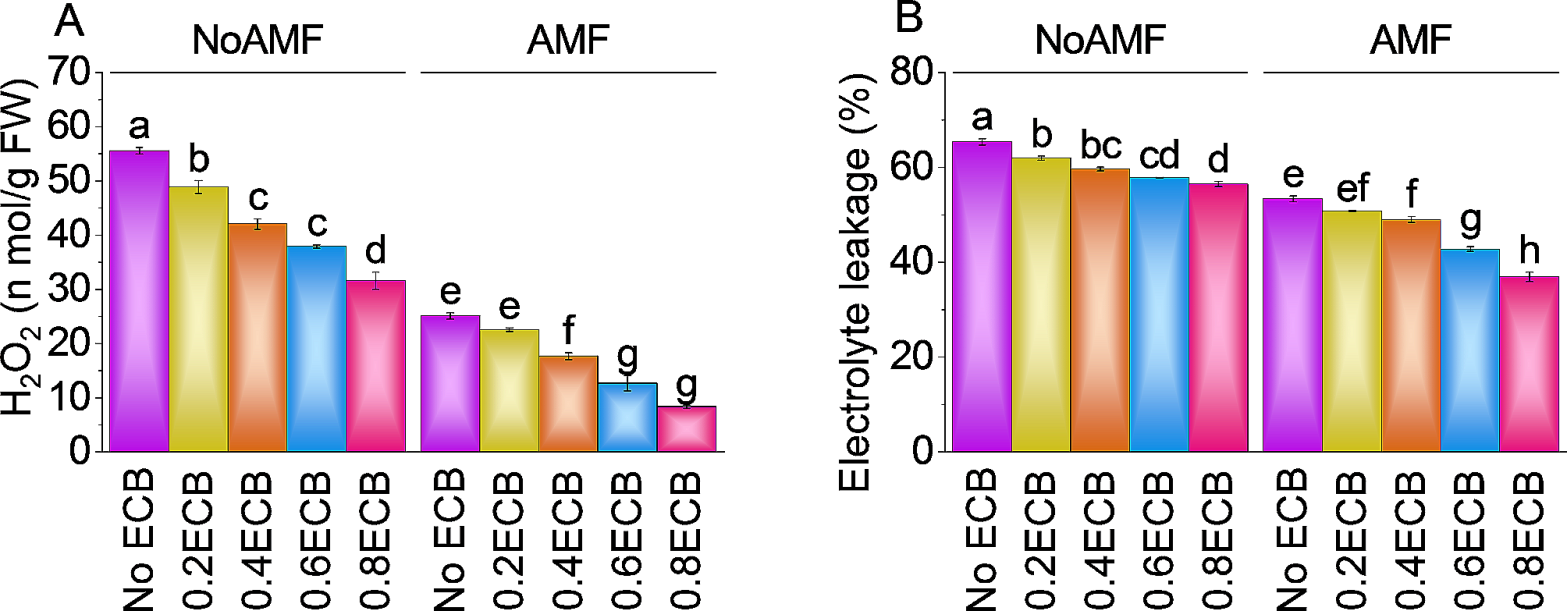
Effect of treatments on H_2_O_2_ (**A**), and electrolytic leakage (**B**) of maize cultivated under NoAMF + 0ECB and AMF + 0ECB. Bars are means of 5 replicates ± SE. Difference letters on bars showed significant changes at *p* ≤ 0.05: Tukey test

The average H_2_O_2_ level without AMF and ECB was ~ 56 nmol/g FW. Without AMF, 0.2ECB treatment decreased H_2_O_2_ by ~ 14%, with a mean value of 48.87 nmol/g FW compared to NoAMF + 0ECB. Compared to NoAMF + 0ECB, adding 0.4ECB reduced H_2_O_2_ by ~ 32% to ~ 42 nmol/g FW. After applying 0.6ECB, mean H_2_O_2_ was ~ 38 nmol/g FW, ~ 47% lower than NoAMF + 0ECB. The highest ECB (0.8ECB), reduced H_2_O_2_ by ~ 76% over NoAMF + 0ECB, with a mean value of ~ 32 nmol/g FW. Over AMF + 0ECB, adding 0.2ECB + AMF decreased H_2_O_2_ by ~ 11%, with a mean value of ~ 23 nmol/g FW. Treatment 0.4ECB + AMF showed a ~ 42% decrease in H_2_O_2_ compared to AMF + 0ECB. At 0.6ECB, mean H_2_O_2_ was ~ 13 nmol/g FW, which was ~ 98% lower than AMF + 0ECB. Applying 0.8ECB + AMF, caused a significant decline in H_2_O_2_ i.e., ~ 201% by showing value of ~ 8 nmol/g FW, over AMF + 0ECB (Fig. 6A).

In NoAMF + 0ECB, electrolyte leakage averaged value was ~ 65%. Adding 0.2ECB without AMF reduced electrolyte leakage by ~ 6%, compared to NoAMF + 0ECB. Compared to NoAMF + 0ECB, adding 0.4ECB caused a decline in electrolyte leakage by ~ 10%. In the case of 0.6ECB electrolyte leakage was ~ 58%, showing a ~ 13% decrease than NoAMF + 0ECB. Result showed that 0.8ECB, resulted in reduction of electrolyte leakage by ~ 16% with a mean value of ~ 57% compared to NoAMF + 0ECB. Treatment 0.2ECB with AMF resulted in minimization of electrolyte leakage by 5.03% over AMF + 0ECB. Adding 0.4ECB + AMF caused a decline in electrolyte leakage to ~ 49%, a ~ 9% decrease over AMF + 0ECB. At 0.6ECB, electrolyte leakage was ~ 43%, which was ~ 25% lower than at AMF + 0ECB. In 0.8ECB + AMF, electrolyte leakage was ~ 37%, showing a ~ 45% decline compared to AMF + 0ECB (Fig. 6B).

### Peroxidase activity, superoxide dismutase activity, catalase activity, and ascorbate peroxidase

**Fig. 7:**
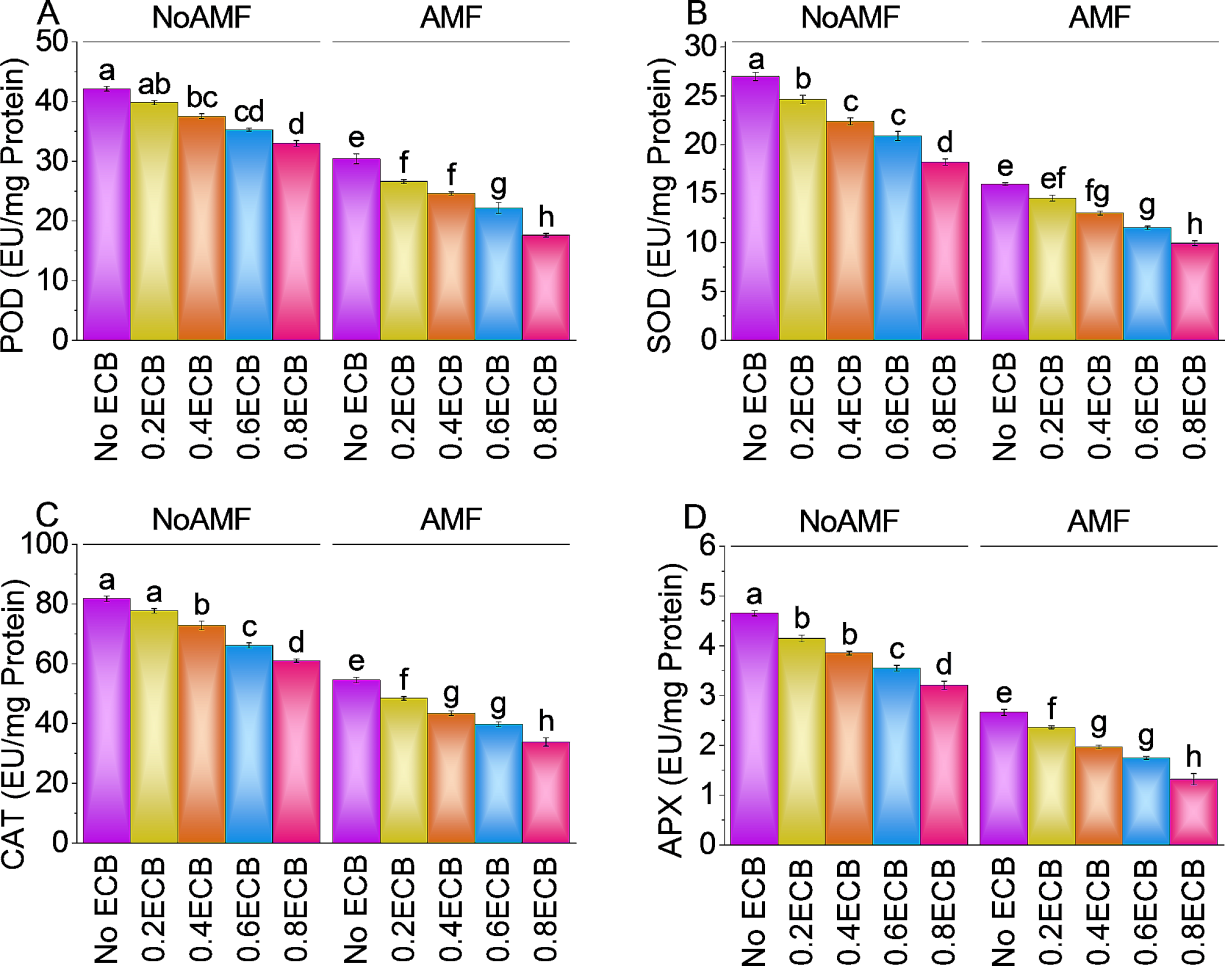
Effect of treatments on stomatal POD (**A**), SOD (**B**), CAT (**C**), and APX (**D**) of maize cultivated under NoAMF + 0ECB and AMF + 0ECB. Bars are means of 5 replicates ± SE. Difference letters on bars showed significant changes at *p* ≤ 0.05: Tukey test

Results showed that 0.2ECB without AMF decreased POD activity by ~ 6% to ~ 40 EU/mg protein compared to NoAMF + 0ECB. Over NoAMF + 0ECB, 0.4ECB reduced POD activity by ~ 12% to ~ 38 EU/mg protein. The average POD activity dcreased ~ 19% from NoAMF + 0ECB to ~ 35 EU/mg protein after applying 0.6ECB. The highest ECB level, 0.8ECB, decreased POD activity by ~ 28% from NoAMF + 0ECB to ~ 33. When AMF was applied without ECB, POD activity averaged ~ 30 EU/mg protein. POD activity decreased ~ 14% averaging ~ 27 EU/mg protein with 0.2ECB + AMF compared to AMF + 0ECB. The POD activity was decline ~ 24% after adding 0.4ECB + AMF. Under 0.6ECB + AMF, POD activity was declined ~ 37% compared to AMF + 0ECB. The POD activity was reduced by ~ 73% at 0.8ECB + AMF, with a mean value of ~ 18 EU/mg protein over AMF + 0ECB (Fig. 7A).

In the absence of AMF and ECB, SOD activity averaged value was ~ 27 EU/mg protein. However, 0.2ECB without AMF decreased SOD activity by ~ 10% compared to NoAMF + 0ECB. Compared to the control group NoAMF + 0ECB, adding 0.4ECB decreased SOD activity by ~ 21%. After applying 0.6ECB, mean SOD activity was ~ 21 EU/mg protein, showing a ~ 29% decrease from NoAMF + 0ECB. Most notably under 0.8ECB, SOD activity was reduced by ~ 48% over NoAMF + 0ECB. However, AMF without ECB had a mean SOD activity of ~ 16 EU/mg protein. It was noted that 0.2ECB + AMF decreased SOD activity by ~ 10% from AMF + 0ECB. Over AMF + 0ECB, 0.4ECB + AMF caused a decline in SOD activity by ~ 23%. The mean SOD activity was 11.51 EU/mg protein at 0.6ECB + AMF, ~ 39% lower than AMF + 0ECB. Results also showed that SOD activity was decreased ~ 61% over AMF + 0ECB under 0.8ECB with AMF (Fig. 7B).

In case of 0.2ECB without AMF, CAT activity was decreased by ~ 5% compared to NoAMF + 0ECB. At 0.4ECB a decreased in CAT activity was ~ 12% than NoAMF + 0ECB. Following 0.6ECB, mean CAT activity was ~ 66 EU/mg protein, which showed a ~ 24% decrease over NoAMF + 0ECB. At 0.8ECB, ~ 34% decline in CAT activity was observed compared to NoAMF + 0ECB. AMF without ECB had a mean CAT activity of ~ 55 EU/mg protein. Applying 0.2ECB + AMF decreased CAT activity by ~ 13% compared to AMF + 0ECB. Compared to AMF + 0ECB, 0.4ECB with AMF reduced CAT activity by ~ 26%. Increasing the ECB level to 0.6ECB decreased CAT activity by ~ 37% compared to AMF + 0ECB. However, CAT activity was significantly reduced by ~ 61% at the highest ECB level i.e., 0.8ECB with AMF, with a mean value of ~ 34 EU/mg protein (Fig. 7C).

Without AMF and ECB, ascorbate peroxidase (APX) activity averaged ~ 5 EU/mg protein. However, 0.2ECB without AMF decreased APX activity by ~ 12% over NoAMF + 0ECB. Additionally, adding 0.4ECB reduced APX activity by ~ 21% compared to NoAMF + 0ECB. After applying 0.6ECB, mean APX activity decreased to ~ 31% compared to NoAMF + 0ECB. In case of 0.8ECB, reduced APX activity i.e., ~ 45% was observed compared to NoAMF + 0ECB. Without ECB, sole application of AMF had a mean APX activity of ~ 3 EU/mg protein. Adding 0.2ECB with AMF decreased APX activity by ~ 13% from AMF + 0ECB, with a mean value of ~ 2 EU/mg protein. In addition, adding 0.4ECB + AMF reduced APX activity by ~ 35% over AMF + 0ECB. Increasing ECB to 0.6ECB decreased APX activity by ~ 53% than AMF + 0ECB. Finally, 0.8ECB combined with AMF, reduced APX activity by ~ 102% in compariosn with AMF + 0ECB (Fig. 7D).

### Glutathione reductase, glutathione, ascorbic acid, and malondialdehyde

**Fig. 8:**
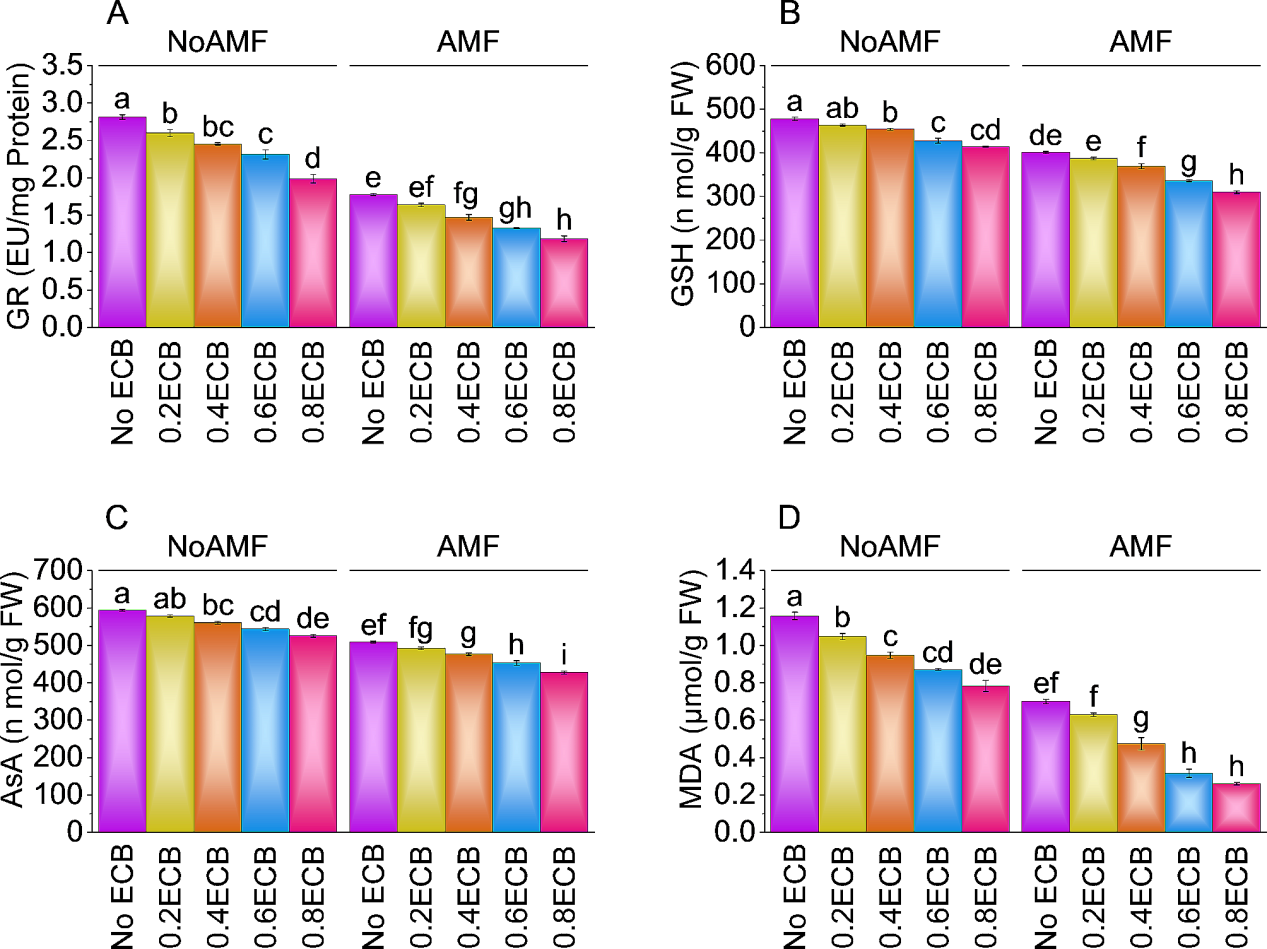
Effect of treatments on stomatal GR (**A**), GSH (**B**), AsA (**C**), and MDA (**D**) of maize cultivated under NoAMF + 0ECB and AMF + 0ECB. Bars are means of 5 replicates ± SE. Difference letters on bars showed significant changes at *p* ≤ 0.05: Tukey test

With no AMF and 0ECB, the mean GR value was ~ 3 EU/mg protein. Compared to NoAMF + 0ECB, 0.2ECB without AMF decreased GR by ~ 8%. Adding 0.4ECB decreased GR by ~ 15% over NoAMF + 0ECB. Results showed that 0.6ECB resulted in ~ 22% decline than NoAMF + 0ECB. Treatment 0.8ECB decreased GR by ~ 42% compared to NoAMF + 0ECB. After 0.2ECB with AMF causing ~ 9% decline in GR than AMF + 0ECB. Compared to AMF + 0ECB, 0.4ECB with AMF reduced GR by ~ 21%. The mean GR value was minimized up to ~ 34% from AMF + 0ECB with 0.6ECB + AMF. It was observed that treatment 0.8ECB + AMF resulted in ~ 50% decrease over AMF + 0ECB (Fig. 8A).

Treatment 0.2ECB without AMF decreased GSH by ~ 3% than NoAMF + 0ECB. Results showed that GSH decreased by ~ 5% after applying 0.4ECB, averaging over NoAMF + 0ECB. Application of 0.6ECB decreased GSH by ~ 12% from NoAMF + 0ECB. However, 0.8ECB caused a decrease in GSH by ~ 15% from NoAMF + 0ECB. Compared to the AMF + 0ECB, 0.2ECB + AMF decreased GSH by ~ 4%, with a mean value of 387.16 nmol/g FW. Over AMF + 0ECB, 0.4ECB + AMF decreased GSH by ~ 9%. Under 0.6ECB, GSH decreased by ~ 19% from AMF + 0ECB. At 0.8ECB + AMF, a decrease of ~ 30% in GSH was observed compared to AMF + 0ECB (Fig. 8B).

In the absence of AMF and ECB, the mean AsA level was ~ 594 nmol/g FW. With a mean value of 579 nmol/g FW, 0.2ECB without AMF decreased AsA by ~ 3% compared to NoAMF + 0ECB. AsA declined ~ 6% with 0.4ECB, averaging ~ 560 nmol/g FW, compared to NoAMF + 0ECB. From NoAMF + 0ECB, applying 0.6ECB caused reduction in AsA (~ 9%). At 0.8ECB AsA decreased ~ 13% compared to NoAMF + 0ECB. When AMF + 0ECB was applied, AsA averaged value was ~ 509 n mol/g FW. Combining 0.2ECB with AMF resulted in a decrease of AsA by ~ 3% over AMF + 0ECB. Over AMF + 0ECB, adding 0.4ECB + AMF caused a decrease in AsA by ~ 7%. The average AsA value declined i.e., ~ 12% over AMF + 0ECB where 0.6ECB + AMF was applied. Under 0.8ECB + AMF, AsA decreased up to ~ 19% that AMF + 0ECB (Fig. 8C).

Adding 0.2ECB without AMF led to a ~ 11% decrease in MDA compared to NoAMF + 0ECB. Results showed that 0.4ECB resulted in a ~ 22% decline in MDA over NoAMF + 0ECB. In the case of 0.6ECB, ~ 33% decrease in MDA was noted over NoAMF + 0ECB. Treatment 0.8ECB significantly reduced MDA levels i.e., ~ 48% compared to NoAMF + 0ECB. AMF without ECB resulted in a mean MDA level of ~ 0.7 µmol/g FW. It was observed that 0.2ECB + AMF caused a decrease of ~ 11% in MDA than control AMF + 0ECB. Additionally, 0.4ECB + AMF resulted in a ~ 47% decline in MDA compared to the control AMF + 0ECB. At 0.6ECB ~ 121% decline in MDA was observed compared to the control AMF + 0EC. However, 0.8ECB + AMF significantly reduced MDA levels by ~ 169% compared to the control AMF + 0ECB (Fig. 8D).

### Cluster plot convex hull, hierarchical cluster plot

The cluster plot convex hull was generated based on the results of a principal component analysis (PCA) performed on the dataset of treatment groups 0ECB, 0.2ECB, 0.4ECB, 0.6ECB and 0.8ECB. The PCA was conducted to reduce the dimensionality of the data and visualize the variation among the treatment groups in a two-dimensional space. The first principal component (PC1) accounted for a significant proportion of the total variation, approximately ~ 98%, while the second principal component (PC2) captured a smaller but still relevant variation, approximately ~ 0.9%. The 0ECB treatment group’s data points were located in the region of PC1 scores ranging from − 9.39634 to 1.28695 and PC2 scores from 0.57994 to -0.74812. Similarly, the data points for the 0.2ECB, 0.4ECB, 0.6ECB, and 0.8ECB treatment groups were plotted with their respective scores on the two principal components.

The data points corresponding to NoAMF samples were in the region of PC1 scores ranging from − 9.39634 to -0.18938 and PC2 scores from 0.57994 to -0.91075. These data points formed a tight cluster, indicating a high level of similarity among samples without Arbuscular Mycorrhizal Fungi. Similarly, the AMF samples were plotted with their respective scores on PC1 and PC2, forming another distinct cluster.

**Fig. 9:**
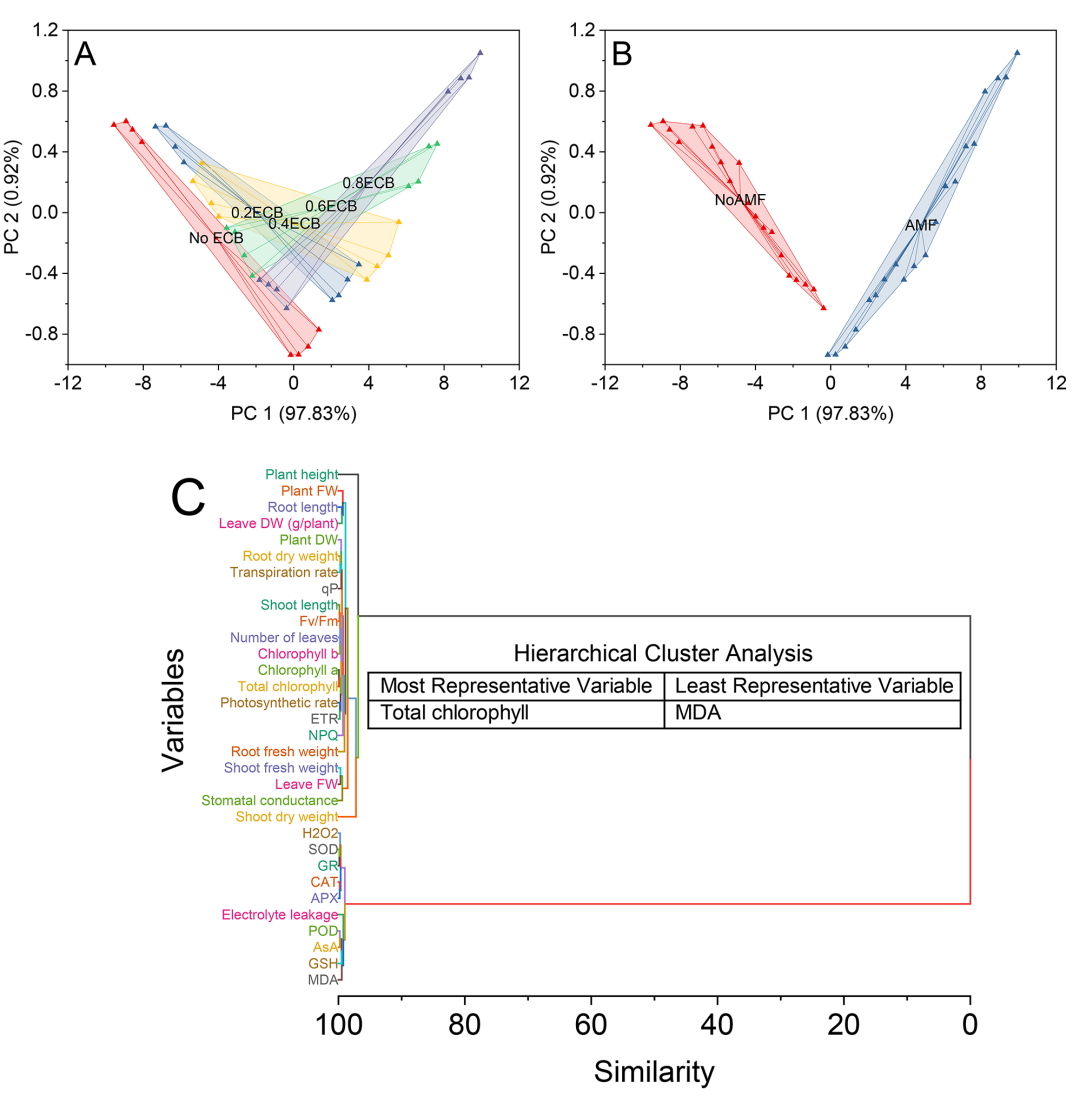
Cluster plot convex hull for treatments (**A**), AMF levels (**B**), and hierarchical cluster plot (**C**) for studied attributes

The clusters revealed meaningful associations, such as chlorophyll a and Total chlorophyll being closely linked with a similarity value of 0.07379, suggesting their interdependence in photosynthetic processes. Similarly, variables like SOD and GR formed a cluster with a similarity value of 0.14812, indicating their potential collaboration as antioxidant enzymes. The analysis also highlighted relationships between shoot length and Fv/Fm, both showing a similarity value of 0.16739, reflecting their involvement in plant growth and photosynthesis. Another significant cluster included CAT and APX with a similarity value of 0.19933, suggesting their role in scavenging reactive oxygen species. Additionally, the clustering revealed distinct groups of variables related to plant weight, such as shoot and root dry weight, both with a similarity value of 0.28984, indicating their impact on overall biomass. Moreover, specific stress-related variables like H_2_O_2_, with a similarity value of 0.25409, and GSH and MDA, with similarity values of 0.52348 and 0.49003, respectively, were each placed in individual clusters, emphasizing their unique roles in plant stress responses (Fig. 9).

## Discussion

### Impact of salinity and EDTA-chelated biochar

Salinity stress significantly impacted maize productivity, evident from the heightened antioxidant activity in the control treatment without any amendments. This decline can be attributed to increased osmotic stress in the rhizosphere, hindering water and nutrient uptake by plants [52]. Dionisio-Sese et al. [53] found an increase in MDA and POD with an increase in salinity stress from 0 to 12 dS m^− 1^. Sairam et al. [54] also reported an increase in antioxidants when salinity stress was increased from 0 to 100 and 200 mM NaCl. However, the application of EDTA-chelated biochar in saline conditions showed an improvement in maize productivity under salinity stress conditions. Akhtar et al. [55] observed a significant enhancement in multiple growth parameters of potato crops under 25 mM NaCl salinity when treated with 5% biochar. Biochar has the capacity to adsorb sodium ions (Na^+^) by increasing potassium (K^+^) content in plants, thus minimize the Na^+^/K^+^ ratios, which play an important role in alleviating salinity stress [54]. Kanwal et al. [56] found that applying 2% biochar led to significant increases in root and shoot length (up to 23% and 11%, respectively). Under 150 mM salt stress, this biochar concentration also maximally improved leaf water potential (16%) and osmotic potential (10%). Additionally, proline content and soluble sugar decreased by 51% and 27%, respectively, with 2% biochar, while superoxide dismutase activity decreased by 15.3%. In another study, Olayinka et al. [57] found improvement in plumule length, radical length, and germination percentage up to 150 mM NaCl concentration with the application of 1.0 mM EDTA. EDTA reduces ionic imbalance stress and toxicity induced by excessive presence of unnecessary ions due to its claw-like structure to bind metals and by creating a stable ring structure [58]. Thus, applications of EDTA-chelated biochar can be helpful in the mitigation of salinity stress and can improve the production of maize.

### AMF role in mitigating salinity stress

AMF form symbiotic relationships with plant roots, extending their hyphal network into the soil [31]. This network effectively increases the root surface area available for nutrient absorption [59]. In saline conditions, where nutrient availability is compromised due to ion imbalances, AMF help in acquiring essential nutrients like phosphorus and nitrogen, supporting crucial plant functions even under stress. AMF also contribute to soil health by enhancing soil structure and organic matter content through their hyphal network. Additionally, they can exclude toxic ions, particularly sodium (Na^+^), from entering the plant system [60]. By preventing excessive Na^ +^ uptake, AMF help in reducing the harmful effects of salinity stress on plants. On the other hand, salinity stress induces osmotic stress, hampering water uptake by plants. AMF assist in osmotic adjustment by accumulating osmolytes, such as proline and soluble sugars, within plant tissues [61, 62]. These compounds regulate osmotic pressure, aiding in water retention and maintaining cellular hydration levels. Consequently, this helps plants maintain turgor pressure and sustain physiological processes [63].

### Synergistic effects of AMF and biochar

The AMF inoculation with EDTA-chelated biochar application showed improved growth and physiology of maize crops in our study compared to EDTA-chelated biochar alone. This might be due to AMF enhancing plant nutrition, improving salt tolerance, and regulating gene expression for stress response, promoting better osmotic adjustment and antioxidant activity [64, 65]. Qin et al. [66] found that AMF-inoculated plants showed a higher net photosynthetic rate, leaf relative water content (RWC), plant height, and osmolyte accumulation under salinity stress in a pot experiment with peanuts. Dastogeer et al. [65] found an increase in the uptake of nitrogen and phosphorus when AMF was inoculated. Our data showed that combined application of AMF and biochar offered synergistic benefits for plant growth. AMF can enhance nutrient uptake and water use efficiency, while biochar improved soil structure, water retention, and nutrient retention. Together, they promote increased crop yield, resilience to salt stress, and sustainable agriculture practices by fostering a more fertile and resilient soil ecosystem. Hammer et al. [67] found that the combined application of biochar and AMF led to increased plant yield, enhanced plant growth, and improved uptake of phosphorus (P) and manganese (Mn) when compared to individual applications in a greenhouse experiment on *Lactuca sativa* under salinity stress. Ndiate et al. [68] found a 14.1% increase in plant height, a 75.7% increase in shoot fresh biomass, a 24.9% increase in root fresh biomass, a 49.5% increase in enzymatic activity, and 30.2–54.8% increase in photosynthetic pigments under salinity stress with the inoculation of AMF and the application of biochar in the wheat crop.

## Conclusions

Salinity stress adversely impacted maize growth, chlorophyll levels, and photosynthetic rates, resulting in higher antioxidant activity. The application of 0.8% EDTA-chelated biochar showed significant improvements in the growth and physiological attributes of maize under salinity stress. Moreover, the effects observed were more pronounced when 0.8% EDTA-chelated biochar and AMF were applied as a combine amendment compared to sole application and control.

## Data Availability

All data generated or analysed during this study are included in this published article.
